# Docosahexaenoic Acid Inhibits Cerulein-Induced Acute Pancreatitis in Rats

**DOI:** 10.3390/nu9070744

**Published:** 2017-07-12

**Authors:** Yoo Kyung Jeong, Sle Lee, Joo Weon Lim, Hyeyoung Kim

**Affiliations:** Department of Food and Nutrition, Brain Korea 21 PLUS Project, College of Human Ecology, Yonsei University, Seoul 03722, Korea; yookyung60@yonsei.ac.kr (Y.K.J.); nutrition15@yonsei.ac.kr (S.L.); jwlim11@yonsei.ac.kr (J.W.L.)

**Keywords:** acute pancreatitis, docosahexaenoic acid, interleukin-6, protein kinase C δ, NF-κB

## Abstract

Oxidative stress is an important regulator in the pathogenesis of acute pancreatitis (AP). Reactive oxygen species induce activation of inflammatory cascades, inflammatory cell recruitment, and tissue damage. NF-κB regulates inflammatory cytokine gene expression, which induces an acute, edematous form of pancreatitis. Protein kinase C δ (PKCδ) activates NF-κB as shown in a mouse model of cerulein-induced AP. Docosahexaenoic acid (DHA), an ω-3 fatty acid, exerts anti-inflammatory and antioxidant effects in various cells and tissues. This study investigated whether DHA inhibits cerulein-induced AP in rats by assessing pancreatic edema, myeloperoxidase activity, levels of lipid peroxide and IL-6, activation of NF-κB and PKCδ, and by histologic observation. AP was induced by intraperitoneal injection (i.p.) of cerulein (50 μg/kg) every hour for 7 h. DHA (13 mg/kg) was administered i.p. for three days before AP induction. Pretreatment with DHA reduced cerulein-induced activation of NF-κB, PKCδ, and IL-6 in pancreatic tissues of rats. DHA suppressed pancreatic edema and decreased the abundance of lipid peroxide, myeloperoxidase activity, and inflammatory cell infiltration into the pancreatic tissues of cerulein-stimulated rats. Therefore, DHA may help prevent the development of pancreatitis by suppressing the activation of NF-κB and PKCδ, expression of IL-6, and oxidative damage to the pancreas.

## 1. Introduction

Acute pancreatitis (AP) is an inflammatory disease associated with abnormal activation and release of digestive enzymes to the pancreas, resulting in auto-digestion of the pancreas and multiple organ dysfunction. It is additionally associated with increased production of cytokines, ultimately leading to deleterious local and systemic effects [1,2,3]. In animal models of pancreatitis, these symptoms have been induced with cerulein, a cholecystokinin (CCK) analogue. Cerulein-induced pancreatitis is one of the best-characterized and widely used experimental animal models of pancreatitis. Supramaximal doses of cerulein result in dysregulation of digestive enzyme production, cytoplasmic vacuolization, death of acinar cells, edema, and infiltration of inflammatory cells into the pancreas [4,5,6].

Although the mechanisms of pathogenesis in AP are not completely clarified, oxidative stress is regarded as a major pathogenic factor [7]. Studies, using experimental models of pancreatitis, indicate that pancreatic oxidative stress occurs during the early stage in the induction of AP [8]. The balance between oxidants and antioxidants is critical for the maintenance of normal function in a biological system. Reactive oxygen species (ROS) exert their pathophysiologic effects by directly attacking lipids [9] and proteins [10] at the local site of generation [11,12]. Additionally, depletion of pancreatic glutathione (GSH) is involved in the early phase of AP [13] and influences the extent of disease severity [14]. The activities of multiple antioxidant enzymes, including glutathione peroxidase, superoxide dismutase, and catalase, and the levels of antioxidant vitamins, decrease the progression of pancreatitis [15,16].

Interleukin (IL)-6 is a pro-inflammatory cytokine that is related in acute-phase responses in inflammation and is produced by a variety of cells, including monocytes/macrophages and endothelial cells, in response to stimulation with endotoxin, IL-1β, and tumor necrosis factor alpha (TNF-α) [17]. Elevated levels of IL-6 have been described in patients with AP and are associated with disease severity [18]. We have previously shown that cerulein-induced activation of NADPH oxidase produces high levels of ROS, which activates the oxidant-sensitive transcription factor NF-κB and induces the expression of IL-6 in pancreatic acinar cells [19]. Inhibiting the expression of IL-6, using treatment with a proliferator of peroxisomes, activated the ligands of receptor-γ (PPAR-γ) and reduced cerulein-induced edema and vacuolization in the rat pancreas [20].

The family of protein kinase C (PKC) plays important roles in signaling to various growth factors, cytokines and hormones. PKCs are serine/threonine kinases comprising 10 isoforms that differ in their structure and regulation [21,22]. In pancreatic acinar cells, four PKC isoforms (α, δ, ε and ξ) have been detected [23,24]. Activation of PKCδ is necessary for mediating the activation of NF-κB, which is involved in the pathogenesis of AP [25].

Docosahexaenoic acid (DHA), one of the ω-3 polyunsaturated fatty acids (PUFAs), is the longest and most unsaturated fatty acid, with 22 carbons and six double bonds (C22:6n-3). DHA exerts antioxidant and anti-inflammatory effects in various cells [26]. We previously reported that DHA inhibited the expression of IL-6 by suppressing the activation of oxidant-sensitive transcription factor activator protein-1 in cerulein-stimulated pancreatic acinar cells [27]. Therefore, we aimed to determine whether DHA inhibits experimental pancreatitis using an in vivo system.

We hypothesized that DHA may inhibit inflammation by suppressing the ROS-mediated activation of PKCδ, NF-κB, and the expression of IL-6 in rats with cerulein-induced AP. We examined the anti-inflammatory effect of DHA, on cerulein-induced AP in rats, by assessing pancreatic edema, the abundance of lipid peroxide (LPO), activity of myeloperoxidase (MPO), expression of IL-6, activation of NF-κB and PKCδ, and histologic changes.

## 2. Materials and Methods

### 2.1. Animals

Thirty seven-week-old male Sprague-Dawley rats (200–250 g) were purchased from Orient Bio (Orient Bio Inc., Seongnam, Kyunggi-Do, Korea). All rats were raised in a climate-controlled room (at 21 ± 2.0 °C and 50 ± 5% humidity) with a 12 h light–dark cycle. All rats were maintained in a specific, pathogen-free facility of Yonsei University. The rats were housed in polypropylene cages on hardwood chip bedding and were provided with food and water ad libitum. The rats were allowed to acclimatize to the laboratory environment for 7 days before the start of the experiments. All experimental procedures were approved by the Institutional Animal Care and Use Committee of Yonsei University (permit No. 201507-328-02). Ten animals were included in each group.

### 2.2. Experimental Design

The experimental design for this rat model of AP, with respect to the injection times and dose of cerulein, was adapted from previous studies [28,29,30]. Briefly, the rats were randomly divided into three groups (*n* = 10 per group): the untreated group (without cerulein), cerulein group (cerulein alone), and cerulein with DHA group (cerulein and DHA). DHA (dissolved in dimethyl sulfoxide (DMSO), 13 mg/kg body weight, administered by intraperitoneal injection (i.p.)), or vehicle (DMSO, 2 mL/kg, i.p.), was administered for 3 days before the cerulein injection. On day 4, cerulein (dissolved in saline containing 0.1% bovine serum albumin (BSA), 50 μg/kg body weight) or vehicle (saline containing 0.1% BSA, 2 mL/kg body weight) was administered every hour for 7 h. The rats were sacrificed 24 h after the first cerulein injection. The untreated group and the cerulein group received DMSO instead of DHA. Pancreatic tissues were collected for histological analysis and immunochemical labeling for PKCδ. Additional tissue samples were homogenized in 10 mM Tris buffer (pH 7.4) for biochemical analyses. The tissue extracts were assessed for the abundance of lipid peroxide (LPO), myeloperoxidase (MPO) activity, and the protein levels of IL-6 and inhibitor of nuclear factor kappa B alpha (IκBα). Total RNA, isolated from the pancreas, was used for the determination of IL-6 mRNA expression. The levels of IL-6 in pancreatic homogenates were determined using enzyme-linked immunosorbent assay (ELISA) and expressed as pg/mg protein. The serum level of IL-6 was determined using ELISA. Additionally, the DNA-binding activity of NF-κB was determined in nuclear extracts of pancreatic tissues.

### 2.3. Measurement of Pancreatic Edema

Pancreatic edema is the primary histological indicator of pancreatic injury and is commonly evaluated by measuring the gain in the water content of the parenchyma [31]. The ratio of pancreas to body weight (g/kg) was utilized to evaluate the degree of pancreatic edema.

### 2.4. Determination of LPO and MPO Activity

Oxidative membrane damage was measured using the level of lipid peroxide (LPO), determined by the thiobarbituric acid reactive substances (TBARS) assay. The level of LPO in tissue extracts was determined using the methods of Ohkawa et al. [32]. Neutrophil infiltration was measured using the myeloperoxidase (MPO) activity assay. MPO activity in tissue extracts was determined using the modified method of Krawisz et al. [33].

### 2.5. Real-Time Reverse Transcription-Polymerase Chain Reaction (RT-PCR)

Total RNA, isolated from the pancreas, was reverse-transcribed into cDNA, using a random hexamer and Moloney-murine leukemia virus (M-MLV) reverse transcriptase (Promega, Madison, WI, USA), with conditions set at 23 °C for 10 min, 37 °C for 60 min, and 95 °C for 5 min. The cDNA was then used for RT-PCR with primers specific for IL-6 and GAPDH. The primers used in this study are described in Table 1. The cDNAs were amplified by 40 cycles of denaturation at 95 °C for 15 s, annealing at 55 °C for 30 s, and extension at 72 °C for 30 s. All the data were normalized to the level of GAPDH.

### 2.6. Western Blot Analysis

Tissue extracts (120 μg protein/lane) were separated using 8–12% sodium dodecyl sulfate-polyacrylamide gel electrophoresis and transferred onto nitrocellulose membranes (Amersham Inc., Arlington Heights, IL, USA) by electro-blotting. The membranes were incubated with the antibodies against phospho-IκBα (Ser32) (1:1000, #2859; Cell Signaling Technology, Danvers, MA, USA), IκBα (1:500, sc-371; Santa Cruz Biotechnology, Dallas, TX, USA), and β-actin (1:500, sc-1615; Santa Cruz Biotechnology) at 4 °C overnight. Next, the membranes were washed using Tris-buffered saline with Tween-20 (TBST). The primary antibodies were detected using horseradish peroxidase-conjugated secondary antibodies, and the proteins were visualized using the ECL detection system (Santa Cruz Biotechnology) according to the manufacturer's instructions. Actin served as the loading control. The protein levels were compared with those of actin. The data were processed and quantified using volume analysis and molecular analysis software, respectively. Protein concentration was determined by the Bio-Rad protein assay (Bio-Rad Laboratories, Hercules, CA, USA).

### 2.7. Enzyme-Linked Immunosorbent Assay (ELISA)

The levels of IL-6 in serum and tissue extracts were determined using ELISA kits (R & D Systems, Minneapolis, MN, USA) according to the manufacturer’s instructions.

### 2.8. Electrophoretic Mobility Shift Assay (EMSA)

EMSA was performed using a method described previously [34]. Briefly, pancreatic tissues were homogenized in a buffer containing 10 mM HEPES, 10 mM potassium chloride (KCl), 0.1 mM ethylenediaminetetraacetic acid (EDTA), 1.5 mM magnesium chloride (MgCl_2_), 0.2 % Nonidet P-40, 1 mM dithiothreitol, and 0.5 mM phenylmethylsulfonyl fluoride; the extracts were centrifuged at 13,000× *g* for 15 min. The resulting pellets were resuspended in nuclear extraction buffer containing 20 mM HEPES, 420 mM sodium chloride (NaCl), 0.1 mM EDTA, 1.5 mM MgCl_2_, 25% glycerol, 1 mM dithiothreitol, and 0.5 mM phenylmethylsulfonyl fluoride, and were then centrifuged at 13,000× *g* for 15 min. The supernatants, containing the nuclear extracts, were collected, and then the total protein level of each sample was quantified using Bradford assay (Bio-Rad Laboratories). An NF-κB gel shift oligonucleotide (AGTTGAGGGGACTTTCCCAGGC; Promega, Madison, WI, USA) was labeled with [^32^P] γ-adenosine triphosphate (Amersham, Piscataway, NJ, USA) using the T4 polynucleotide kinase (Promega). The end-labeled probe was purified from an unincorporated [^32^P] γ-adenosine triphosphate using a Bio-Rad purification column (Bio-Rad Laboratories) and recovered in Tris-EDTA buffer. Nuclear extracts (2 μg) were incubated with a buffer containing the ^32^P-labeled NF-κB consensus oligonucleotide at 15–25 °C for 30 min and electrophoretically separated on a nondenaturing acrylamide gel. The gels were dried at 80 °C for 2 h and exposed to radiography film for 24 h at −70 °C with intensifying screens.

### 2.9. Immunohistochemical Analysis

Pancreatic tissue sections (at the thickness of 4 μm) were deparaffinized in xylene for 15 min, rehydrated using an ethanol gradient, and heated in 10 mM sodium citrate buffer (pH 6.0) in a microwave oven at 95 °C for 5 min for antigen retrieval. Endogenous peroxidase activity was blocked by immersing the slides in a peroxidase-blocking buffer (3% hydrogen peroxide, Duksan hydrogen peroxide 3095, Duksan Lab., Seoul, Korea) for 10 min at 15–25 °C. After antigen retrieval, the tissue sections were immersed in blocking buffer (5% BSA) for 1 h at room temperature. The tissue sections were then incubated with the primary antibody against PKCδ (1:100, ab182126, Abcam, Cambridge, UK) at room temperature for 2 h. The tissue sections were then rinsed in TBS and incubated with a secondary antibody (horseradish peroxidase (HRP)-labeled anti-rabbit, Rockland Immunochemicals Inc. Limerick, PA, USA) for 20 min at room temperature. The tissue sections were again washed in TBS, and the bound peroxidase was detected by incubating the tissue sections in 3,3′-diaminobenzidine (DAB) substrate for 3 min at room temperature. The tissue sections were then washed in distilled water and the nuclei were counterstained with Mayer’s hematoxylin for 1 min, followed by two rinses in distilled water. The sections were dehydrated by serial immersion, for 1 min each, in 70% ethanol and 95% ethanol, followed by 2 min each in two changes of 100% ethanol and two changes of xylene. A coverslip was mounted over each tissue section using Pertex mounting medium.

### 2.10. Histological Observation

One portion of the pancreas was fixed overnight at 4 °C in freshly prepared formaldehyde (Sigma-Aldrich, St. Louis, MO, USA) in PBS, pH 7.4. The tissue was then embedded in paraffin, sectioned 0.18 mm long and 5 micron thick and processed with hematoxylin and eosin (Sigma-Aldrich) using standard procedures. Multiple randomly chosen microscopic fields, from 10 rats in each treatment group, were examined for edema development and neutrophil infiltration by a pathologist blinded to the treatment. The severity of pancreatitis was documented by scoring the extent of edema, leukocyte infiltration, and necrosis of acinar cells. Each criterion was graded on a scale of 0–4 (normal to severe) as previously described [34,35].

### 2.11. Statistical Analysis

All values were expressed as means ± S.E. for the 10 rats in each group. Statistical significance was assessed using analysis of variance, followed by Newman–Keuls post hoc test [36]. A *p*-value < 0.05 was considered statistically significant.

## 3. Results

### 3.1. DHA Reduced Cerulein-Induced Pancreatic Edema in Rats

**Figure 1:**
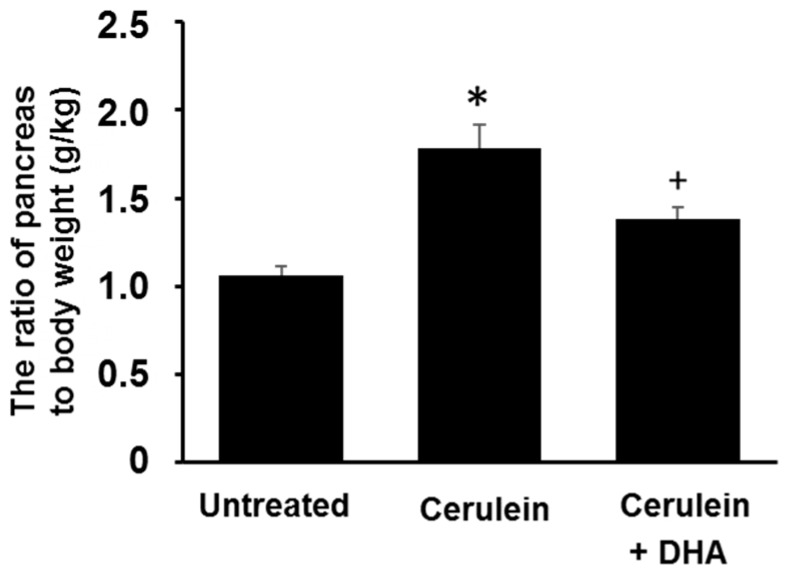
Effect of docosahexaenoic acid (DHA) on pancreatic edema in rats. The ratio of pancreas to body weight was used as an indicator of pancreatic edema. Values are mean ± S.E. for the 10 rats in each group. * *p* < 0.05 vs. untreated group (without cerulein), ^+^ *p* < 0.05 vs. cerulein group (cerulein alone).

The ratio of pancreas to body weight (g/kg) was measured to evaluate the degree of pancreatic edema. The ratio of pancreas to body weight was increased approximately two-fold in the cerulein-treated group compared with that of the untreated group. Pretreatment with DHA for three days before the cerulein injection attenuated the formation of cerulein-induced pancreatic edema (Figure 1).

### 3.2. DHA Reduced the Abundance of LPO and Activity of MPO in Pancreas of Cerulein-Stimulated Rats

**Figure 2:**
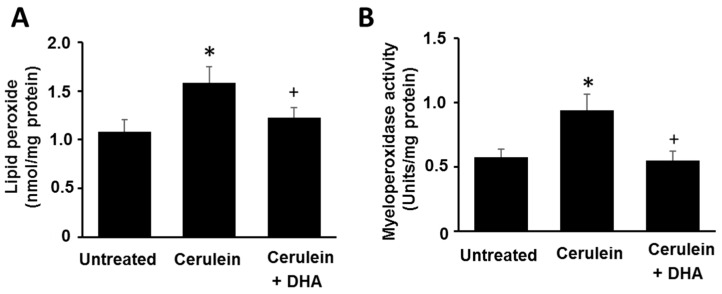
Effect of DHA on the abundance of lipid peroxide (LPO) and activity of myeloperoxidase (MPO) in the pancreas. (**A**) The abundance of lipid peroxide is expressed as nmol/mg protein; (**B**) Myeloperoxidase activity is expressed as units/mg protein. Values are mean ± S.E. for the 10 rats in each group. * *p* < 0.05 vs. untreated group (without cerulein), ^+^ *p* < 0.05 vs. cerulein group (cerulein alone).

As shown in Figure 2, cerulein increased the level of LPO, a marker of oxidative damage (A), and activity of MPO, an indicator of tissue-associated neutrophil accumulation (B), in pancreatic tissues. DHA suppressed the cerulein-induced increase in the level of LPO and activity of MPO in the pancreas. These results indicate that DHA may attenuate pancreatic inflammation by suppressing cerulein-induced neutrophil infiltration and oxidative damage in the pancreas.

### 3.3. DHA Inhibited Cerulein-Induced Histopathologic Changes in Rat Pancreas

**Figure 3:**
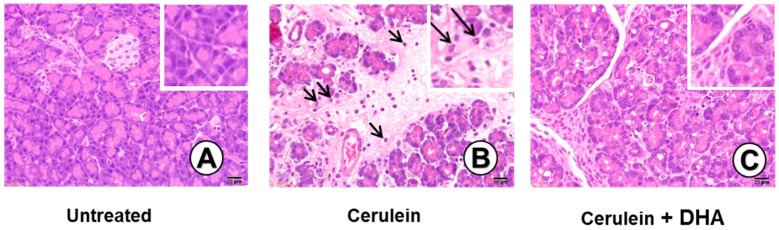
Effect of DHA on cerulein-induced histopathological changes in the pancreas. Images (**A**–**C**) display representative examples of pancreatic tissues. (**A**) Normal pancreatic tissue is seen in the untreated group; (**B**) Abnormal architecture, including inflammatory cell infiltration (arrow) and edematous lesion, are observed in cerulein-treated group; (**C**) Reduced edematous lesions are observed in the group treated with cerulein and DHA. Hematoxylin & eosin (H&E) stain, magnification: 400×; Scale bar, 20 μm (magnification in each top right panel is 800×).

**Figure 4:**
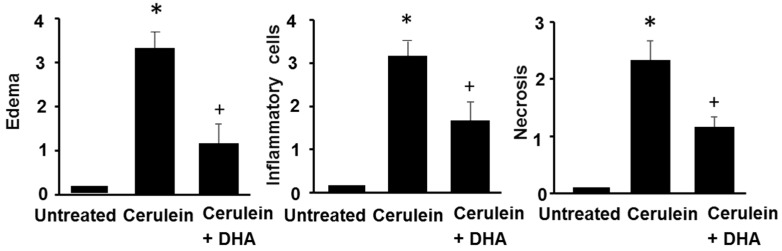
The effect of DHA on histological scores in pancreatic injury. H&E-stained sections were evaluated for edema, inflammatory cell infiltration, and necrosis of acinar cells. Values are mean ± S.E. for the 10 rats in each group. * *p* < 0.05 vs. untreated group (without cerulein); ^+^ *p* < 0.05 vs. cerulein group (cerulein alone).

Under a microscopic examination, the pancreas in the untreated group were morphologically intact, whereas those in the cerulein-treated control group showed severe interstitial edema and infiltration of neutrophils into the pancreas (Figure 3A,B). However, treatment with DHA substantially reduced the abnormal architecture such as interstitial edema and inflammatory cell infiltration (Figure 3C). Pathological scoring of the pancreatic damage revealed cerulein-induced edema, inflammatory cell infiltration, and necrosis of acinar cells (Figure 4). Treatment with DHA markedly reduced the severity of AP.

### 3.4. DHA Reduced IL-6 Expression in the Pancreas of Cerulein-Induced Rats

**Figure 5:**
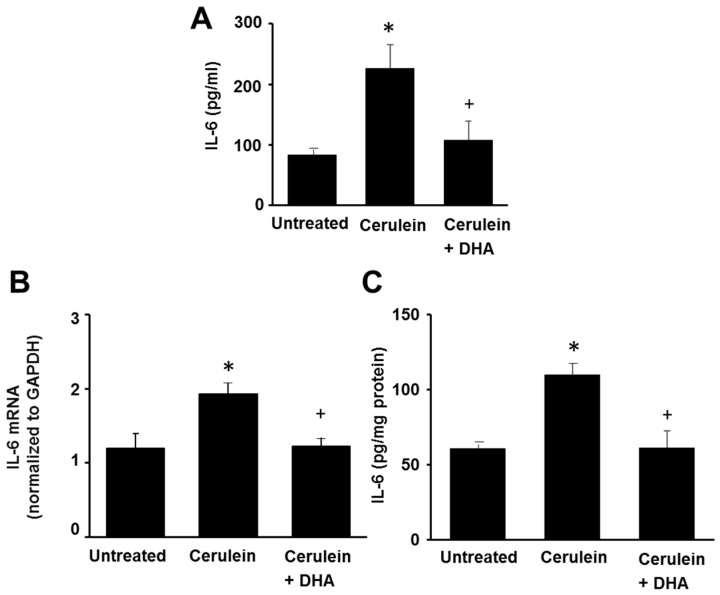
The effect of DHA on the serum level of IL-6, and the levels of IL-6 mRNA and protein in the pancreas. (**A**) The level of IL-6 in the serum was determined using ELISA (**B**) RT-PCR was performed on reverse-transcribed RNA isolated from the pancreatic tissue. The mRNA level of IL-6 was normalized to that of GAPDH; (**C**) The protein level of IL-6 in the pancreas was determined using ELISA and expressed as pg/mg protein. Values are mean ± S.E. for the 10 rats in each group. * *p* < 0.05 vs. untreated group (without cerulein), ^+^ *p* < 0.05 vs. cerulein group (cerulein alone).

Figure 5 shows that cerulein increased the serum level of IL-6 and induced mRNA and protein expression of IL-6 in the pancreas. DHA inhibited the cerulein-induced increase of IL-6 levels in the serum and pancreas. These results are similar to the effect of DHA on the development of pancreatic edema, abundance of LPO, activity of MPO, and histologic damage. Therefore, IL-6 expression may reflect the pancreatic oxidative damage induced by cerulein. DHA may prevent pancreatic injury by suppressing the expression of IL-6 in cerulein-induced pancreatitis.

### 3.5. DHA Inhibited Cerulein-Induced Phosphorylation and Degradation of IκBα and Activation of NF-kB in the Rat Pancreas 

**Figure 6:**
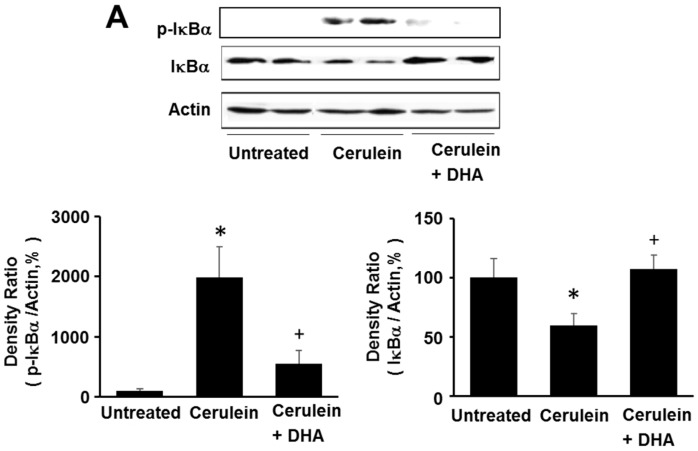

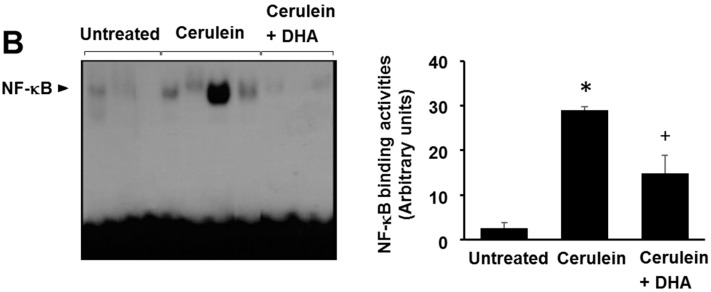
Effect of DHA on the levels of phospho-IκBα (p-IκBα), total IκBα, and NF-κB-DNA binding activity in the pancreas. (**A**) Western blotting was performed using antibodies against p-IκBα and total IκBα. Protein levels of p-IκBα and total IκBα were compared with that of the loading control actin and expressed as a percentage ratio of the band density; (**B**) Electrophoretic mobility shift assay (EMSA) for the activity of NF-κB was performed using nuclear extracts from pancreatic tissues. Each lane represents data for an individual animal. Values are mean ± S.E. for the 10 rats in each group. * *p* < 0.05 vs. untreated group (without cerulein), ^+^ *p* < 0.05 vs. cerulein group (cerulein alone).

Here, we investigated the effect of DHA on cerulein-induced activation of NF-κB by determining the phosphorylation and degradation of IκBα, and NF-κB-DNA binding activity, in the pancreas. As shown in Figure 6A, the levels of phosphorylated IκBα increased; however, the total levels of IκBα were decreased by cerulein. The phosphorylation and degradation of IκBα, induced by cerulein, was inhibited by DHA. Additionally, DHA suppressed the cerulein-induced activation of NF-κB in the pancreas (Figure 6B). These results strongly suggest that by inhibiting NF-κB activation, DHA may suppress cerulein-induced expression of inflammatory mediators.

### 3.6. DHA Suppressed Cerulein-Induced Activation of PKCδ in Rat Pancreas

**Figure 7:**
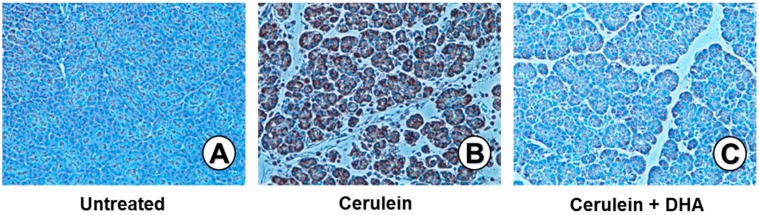
Effect of DHA on the level of PKCδ in the pancreas. Immunohistochemical analysis was performed using an anti-PKCδ antibody. (**A**) A scant signal for PKCδ was detected in the untreated group; (**B**) An intense signal for PKCδ was detected in the cerulein-treated group; (**C**) Few cells, positive for PKC-δ, were detected in the group treated with cerulein and DHA. Magnification: 200×.

Figure 7 shows immunohistochemical analysis for the expression of PKCδ in the pancreas. Scant signaling for PKCδ was observed in the pancreas of the untreated group (Figure 7A). Significant levels of PKCδ were detected in the cytoplasm and membrane of pancreatic acini in the cerulein-treated group (Figure 7B). However, only a few cells, positive for PKCδ, were observed in the group treated with cerulein and DHA (Figure 7C). These results suggest that DHA suppresses the cerulein-induced expression of PKCδ in the pancreas.

## 4. Discussion

In this study, we found that an intraperitoneal injection of DHA inhibited the cerulein-induced development of pancreatic edema and reduced the abundance of LPO, activity of MPO, and expression of inflammatory cytokine IL-6 in the pancreas. DHA inhibited cerulein-induced activation of NF-κB and PKCδ in the pancreas. Furthermore, cerulein-induced histologic changes, such as edematous lesions and inflammatory cell infiltration into the pancreatic tissue, were suppressed by DHA. These results demonstrate the inhibitory effect of DHA on cerulein-induced AP. In this model, DHA may prevent AP by inhibiting the oxidative stress-mediated activation of NF-κB and PKCδ, inflammatory cytokine IL-6, and resulting tissue damage in the rat pancreas.

Fish oil, and its components, ω-3 PUFAs, have shown anti-inflammatory properties and, thus, play a beneficial role in the prevention and treatment of inflammatory disorders such as cardiovascular diseases [37,38]. DHA, the longest and most unsaturated ω-3 PUFA, possesses the most robust antioxidant and anti-inflammatory activity among ω-3 PUFAs [39]. In this study, we confirmed the antioxidant and anti-inflammatory effects of DHA against cerulein-induced pancreatitis. DHA decreased the cerulein-induced increase in LPO abundance and expression of the pro-inflammatory cytokine IL-6, which is a key immune response mediator in pancreatic inflammation [40]. Additionally, DHA decreased cerulein-induced activation of PKCδ and NF-κB in the pancreatic tissue. These results indicate that DHA inhibited the expression of IL-6 via downregulation of PKCδ, and inhibition of NF-κB, in the pancreas of cerulein-stimulated rats.

DHA alters the redox status of cells; this effect is dependent on the concentration of DHA used to treat the cells. Véricel et al. [41] reports that a high concentration of DHA (>100 μM) induced oxidative stress in human platelets, whereas a low level of DHA inhibited oxidative stress. Our previous study demonstrated that low concentrations of DHA (20–50 μM) decreased the levels of ROS by upregulating the antioxidant enzyme catalase in pancreatic acinar cells [42]. However, a high concentration of DHA (>100 μM) increased ROS production and apoptosis in cancer cells including pancreatic cancer cells [43,44]. Trépanier et al. [45] reports that daily intraperitoneal injections of 50 mg/kg DHA increased the levels of unesterified DHA up to 1 μM in the serum. Therefore, intraperitoneal injections of 13 mg/kg DHA, used in the present study, may maintain unesterified DHA in the serum at a level less than 1 μM. This low level of DHA may contribute to the inhibition of oxidative stress in the pancreas.

Several studies show that the levels of serum amylase, lipase and IL-6 are significantly increased in cerulein-stimulated mice and rats, and are reduced by the 5-HT3 receptor antagonist ondansetron [46], propylene glycol alginate sodium sulfate [47], and certolizumab, a pegylated monoclonal antibody to TNF-α [48]. However, no study has yet determined the effect of DHA on the serum levels of amylase, lipase and IL-6 in the models of cerulein-induced pancreatitis. In this study, we found that DHA reduced the serum level of IL-6, which was increased in rats by stimulation with cerulein. To confirm the anti-inflammatory effect of DHA on cerulein-induced pancreatitis, serum levels of amylase and lipase should be monitored in cerulein-stimulated rats, treated or untreated with DHA, for further study.

Cerulein is a physiological agonist that activates phospholipase C, leading to the generation of inositol trisphosphate and diacylglycerol; trisphosphate, then, releases Ca^2+^ from the intracellular stores and diacylglycerol activates PKC [49]. Several reports show that calcium signaling mediates ROS production in several cell lines [50,51]. We previously demonstrated that treatment with a Ca^2+^ chelator, BAPTA-AM, inhibited cerulein-induced ROS production in pancreatic acinar cells [52]. Moreover, cerulein activated NADPH oxidase to produce high levels of ROS in the pancreatic acinar cells [19]. These studies demonstrate that cerulein-induced increase in calcium may directly activate PKC and induce activation of NADPH oxidase to produce ROS in the pancreas.

Oxidant-sensitive transcription factor NF-κB can be activated by ROS, PKC and intracellular Ca^2^ in pancreatic acinar cells [53,54]. A specific PKCδ translocation inhibitor (δi) and genetic deletion of PKCδ inhibit CCK-8-induced NF-κB activation in pancreatic acinar cells [55]. These studies suggest that PKCδ is an upstream regulator of NF-κB activation in cerulein-treated pancreatic acinar cells. However, treatment of pancreatic acini with GF109203X, a selective cell-permeant inhibitor of PKCδ, inhibited AP but not the generation of ROS [56]. Therefore, the production of ROS by NADPH oxidase, and activation of PKCδ, may be independent pathways in cerulein-stimulated pancreatic acinar cells. Our study demonstrates that both ROS and PKCδ activate NF-κB to induce the expression of inflammatory cytokine IL-6 in the pancreas.

NF-κB is a central molecule that links the initial acinar injury to systemic inflammation and perpetuates the inflammation [57]. Activation of PKCδ is involved in the mediation of AP [25]. Therefore, the inhibitory effect of DHA on the activation of NF-κB and PKCδ may, in turn, inhibit the development of AP. These results are supported by studies showing that DHA inhibits inflammation via suppression of PKCδ activation in various cell types [58,59].

Many studies show that NF-κB is activated early in acinar cells during AP and increases the expression of multiple pro-inflammatory genes. Pharmacological inhibition of NF-κB ameliorates AP [60,61,62,63]. Additionally, pancreatitis has been induced by pancreatic expression of p65 using adenoviral-mediated gene transfer and by transgenic expression of active IκB kinase 2 in the pancreas [64,65]. These studies strongly suggest that elevated NF-κB activity increases the severity of pancreatitis. However, Algul et al. [66] shows that mice, with genetic elimination of NF-κB signaling in pancreatic cells, developed a more severe pancreatitis compared with those with wild-type NF-κB. This study demonstrates that NF-κB activity is essential for protecting the pancreas. Under genetic manipulation, in which NF-κB is completely eliminated, cells are damaged. A basal level of NF-κB activity is required for cell survival. However, overwhelming activation of NF-kB leads to increased expression of inflammatory cytokines, such as IL-6, which contributes to the development of pancreatic inflammation.

To activate NF-κB, the inhibitory protein IκBα needs to be phosphorylated by IκBα kinase. Phosphorylated IκBα is subsequently polyubiquitinated and degraded by the 26S proteasome. Therefore, it is necessary to determine the levels of phosphorylated IκBα and IκBα kinase in cerulein-stimulated pancreatic acinar cells. In this study, cerulein increased the levels of phosphorylated IκBα, but decreased those of total IκBα; this effect, in the rat pancreas, was inhibited by treatment with DHA. These results demonstrate that cerulein activates NF-κB, and DHA inhibits cerulein-induced activation of NF-κB, in the rat pancreas.

Pancreatic edema formation is the histological indicator of pancreatic injury [34,35]. MPO activity is considered as histopathological grading for tissue-associated neutrophil accumulation [33]. Since cerulein induced infiltration of neutrophils into the pancreas, MPO activity increased in cerulein-stimulated pancreas. Cerulein directly produce ROS by activating NADPH oxidase in pancreatic acinar cells and ROS are produced by the inflammatory cells; ROS induce peroxidation of lipid to increase LPO level in pancreas.

We hypothesized that DHA may protect the pancreas against oxidative stress and inflammation. In this study, DHA suppressed cerulein-induced increases in the development of edema, abundance of LPO, activity of MPO, induction of IL-6, and activation of NF-κB and PKCδ in the pancreatic tissues of cerulein-stimulated rats. Additionally, histological changes, such as neutrophil infiltration and edematous lesions, induced by cerulein, were suppressed by treatment with DHA. These results support our hypothesis and demonstrate the preventive effect of DHA against cerulein-induced AP.

Recently, we found that DHA acts as an agonist of the peroxisome proliferator activated receptor γ and induces catalase expression in pancreatic acinar AR42J cells [42]. Thus, DHA reduces ROS levels and inhibits the ROS-mediated activation of Janus kinase (JAK) 2/signal transducer and activator of transcription (STAT)3 and IL-6 expression in cerulein-stimulated pancreatic acinar cells. This finding may support our results, showing that DHA inhibited the expression of IL-6, induced by cerulein, in the rat pancreas.

In this study, DHA was administered to the rats prior to stimulation with cerulein. Therefore, consumption of DHA-rich foods may prevent pancreatic inflammation by reducing oxidative stress. To determine whether DHA is useful for treating pancreatitis, further studies should investigate the effect of administering DHA simultaneously with cerulein or after stimulation with cerulein. These studies may indicate whether administration of DHA is beneficial after the induction of AP and may offer a clinical use of DHA for treating patients with AP.

## 5. Conclusions

This study demonstrates the inhibitory effect of DHA on cerulein-induced AP. DHA inhibited oxidative stress and pancreatic inflammation by suppressing the activation of NF-κB and PKCδ, induction of IL-6, and oxidative damage in the pancreas. Therefore, DHA may be beneficial for preventing the development of pancreatitis by suppressing the inflammatory signaling and oxidative tissue injury in the pancreas.

## Figures and Tables

**Table 1:** Primers for RT-PCR.

Target Gene	Primer	Sequence (5′-3′)
*GAPDH*	Forward primer	GAAGGTGAAGGTCGGAGT
	Reverse primer	GAAGATGGTGATGGGATTC
*IL-6*	Forward primer	GAGAGGAGACTTCACAGAGGATACCAC
	Reverse primer	ACCACAGTGAGGAATGTCCACAA
